# Doxycycline: From Ocular Rosacea to COVID-19 Anosmia. New Insight Into the Coronavirus Outbreak

**DOI:** 10.3389/fmed.2020.00200

**Published:** 2020-05-08

**Authors:** Chiara Bonzano, Davide Borroni, Andrea Lancia, Elisabetta Bonzano

**Affiliations:** ^1^Eye Clinic, DiNOGMI, University of Genoa and IRCCS San Martino Polyclinic Hospital, Genoa, Italy; ^2^Cornea Unit, Royal Liverpool University Hospital, Liverpool, United Kingdom; ^3^Department of Radiation Oncology, IRCCS San Matteo Polyclinic Foundation, Pavia, Italy; ^4^PhD School in Experimental Medicine, University of Pavia, Pavia, Italy

**Keywords:** COVID-19, doxycycline, coronavirus (CoV), SARS – CoV, anosmia

## Introduction

Coronavirus Disease 19 (COVID-19) caused by Severe Acute Respiratory Syndrome-Coronavirus-2 (SARS-CoV-2) it usually manifests with respiratory symptoms (1).

Similarly, to other human respiratory Coronaviruses (HCoV), it seems to have a neuroinvasive and neurotropic activity (1, 2). In the retrospective case series study conducted by Mao et al. three categories of neurological symptoms COVID19-related included central nervous system (CNS) manifestations, peripheral nervous system (PNS) symptoms and musculoskeletal disorders (2).

Hyposmia has been reported as a possible peripheral nervous system (PNS) symptom caused by COVID-19 infection (2).

In our experience, the smell alteration (hyposmia, anosmia) seems to be one of the first manifestations of COVID-19 disease, with or without the loss of taste (dysgeusia). Sometimes it remains the only symptom; more often, it comes with fatigue, fever, and cough.

We provide a commentary on how COVID-19 could affect the sense of smell and the reason why doxycycline (Dox) could play a role in its recover.

## Anosmia

The three leading causes of loss of smell reported in the literature are head trauma, chronic sinonasal inflammation and upper respiratory tract viral infections (3, 4). Anosmia is one between numerous olfactory disorders, but its mechanism is not clearly defined (3).

Post viral temporary chemosensory dysfunction after a common cold is widely reported (3, 5, 6).

The swelling of the mucosa in the olfactory cleft it seems to be cause of the transient olfactory and taste loss typically reported during the common cold. It usually leads to a conductive post-viral loss of smell, and it usually appears 3 months after the upper respiratory tract infection (3).

The olfactory neuroepithelium represents an important immunological barrier within the nasal cavity exposed to the external environment, and thus it is subject to both exogenous insults and endogenous host defense responses (7).

In the pathogenesis of chronic rhinosinusitis (CRS)-associated olfactory loss, interferon (IFN)γ- signaling pathways may play a pivotal role in orchestrating immune system function. They are able to modulate inflammatory response and pattern-recognition receptors expressed by the innate immune system during infection (7). Therefore, they support an inflammatory process underlying the olfactory impairment CRS-linked (8). As evidence of this (CRS)-associated olfactory dysfunction is relatively rapidly reversed with systemic corticosteroids (9).

On the other side, some post-viral sense of smell impairment may be partly independent of nasal congestion, thus explaining oxymetazoline failure in improving olfaction (10).

Therefore, it has been suggested that HCoV, thanks to their neuroinvasive, neurotropic, and neurovirulent properties may be able to induce neuronal impairment (11).

### “Speed and Simplicity” of SARS-CoV-2

Entering into the respiratory tract, all HCoV invade and infect intra-luminal macrophages and epithelial cells. HCoV belong to Coronaviridae, enveloped non-segmented, single-stranded, positive-sense RNA viruses (+)ssRNA. Viral spike (S) proteins manage their cell entry program. They act by binding cell-surface receptors and facilitating the fusion of the virus-cell membrane (12). The spike protein is the key of coronaviruses tropism (12). These S proteins are organized in trimers that end up on the virion in a “corona” way giving it the characteristic crown-like look which seems to play a significative role in viral infection and pathogenesis (12). Similarly to SARS-coronavirus, SARS-CoV-2 seems able to enter in the respiratory epithelium (RE) by binding the human ACE2 receptor. Recombinant S protein has been shown to interact with recombinant ACE2 protein (13). S proteins are the major antigenic determinant managing host immune networks, inhibiting antibodies and the immunity response against the virus by inactivating IFN-α and IFN-ß (12, 14). It is well-established the pivotal role of IFN to protect most tissues from viral pathogenicity. The speed and simplicity of SARS-CoV-2 are typical. Host survival in the presence of the viral infection depends on the efficacy of its IFN system; as a matter of fact, virus survival is linked to its capacity to replicate and spread in the host, by carrying out mechanisms of evasion or subversion of the host IFN response (15).

### New Insights Into the Doxycycline Activity

IFNα/β signaling plays a protective role in reducing the virus spread and modulating T cell non-cytolytic antiviral response in limiting viral load. Moreover, some RNA-viruses have developed mechanisms to counteract innate host defense to establish productive infections in their hosts. This is the case of an RNA virus, the vesicular stomatitis virus (VSV) (16).

Retinoic acid-inducible gene I (RIG-I) and melanoma differentiation-associated gene-5 (Mda-5), seem to have an important role in the recognition of RNA viruses. In particular, it has been shown that immune signaling by RIG-I is involved in the generation of IFN-α/β following VSV infection. Under Dox treatment, cells released high levels of RIG-I proteins eliciting autonomous IFN response, thereby inhibiting viral infection *in vitro* (17).

In another RNA virus, the Respiratory Syncytial Virus (SRV), viral proteins inhibit IFN-α and IFN-β to establish infection (18), and it has been reported a higher expression of interferon-induced protein only after minocycline administration. This suggests an increasing innate immune response supported by tetracycline and the following RSV inhibition (19).

The second-generation tetracycline Dox has an anti-inflammatory and broad spectrum antimicrobial activity (20, 21).

In 1967, Dox was first approved by the FDA (20). It has minimal side effects and it is routinely prescribed for acne and rosacea. Dox is characterized by a ~100% oral absorption and a prolonged serum half-life (18–22 h) (22).

In ophthalmology, Dox is usually administered in patients affected by ocular rosacea and posterior blepharitis (23). The Dox recommended dose is 40 mg modified release once daily, which could be replaced by minocycline 100 mg, based on patient tolerance or particular requirements (24).

The rationale in its administration is proteolysis inhibition promoted by matrix metalloproteinases (MMPs) (23, 25). MMPs are involved in the regulation of chemical and biological process likes vascular remodeling and angiogenesis (26), so Dox also has anti-angiogenic properties. (27) It regulates cytokines and diminishes neutrophil chemotaxis too (28).

Besides its well-known use in treating bacterial infections, some studies in the literature report that Dox possesses a broad activity against viral infection too (29–31).

The first who described the Dox antiviral effect was Sturtz in 1998 (29), and this suggestion has been confirmed in several followed-up studies. (16, 32, 33)

Topno et al. demonstrated that Dox could interfere with the virion's replication, affecting its structure and causing inhibition of Japanese encephalitis virus-induced pathogenesis *in vitro* (32). The same observation is also reported in a study regarding VSV infection (16) and against the chikungunya virus (CHIKV) (33), suggesting that Dox might interfere with viral replication by aiming proteins essential for these viruses for a successful infection. As proof of that, computational literature reports the Dox ability to bind CHIKV cysteine protease (33), and to exert a significant inhibitory effect on DNV NS2B-NS3 serine protease *in vitro* (30); both these proteases proved to be able to catalyze viral polyproteins cleavage during infection. Moreover, some studies with (+)ssRNA, Dengue virus (DNV), have demonstrated that Dox inhibits virus plaque assembly by interfering with the viral envelope conformational changes needed for virus entry (30). In both CHIKV and DNV, Dox seems to have the ability to bind virus envelop inhibiting viral entry into the cultured cells (30, 33).

Dox proved to be able to markedly decreased the virus-induced cytopathic effect (CPE) and significantly affect viral replication in a dose-dependent manner when used against Porcine Reproductive And Respiratory Syndrome virus (PRRSV) infection in cultured cells (31). Virus mRNA levels were strikingly reduced also in VSV-infected cells in response to Dox; both virus titers and the CPE of VSV infection were significantly influenced by Dox administration in a dose dependent manner (16).

## Discussion

Being the olfactory neural system able to regenerate throughout life, it can explain why the recovery of olfaction is common (34).

From our observation, anosmia affected mostly young adults rather than elderly patients, confirming existing findings in the literature (35, 36). It shows up more or less 6 days after fever, cough and muscle aches, but it can be the first and only symptom in many patients, with no mucosal swelling of the olfactory cleft, and that's why we hypothesize that it could be a possible PNS symptom as suggested (2). Among patients affected by PNS symptoms linked to COVID-19, the most common referred were hyposmia, hypogeusia, followed by neuralgia (2). Respiratory viruses such as rhinovirus and parainfluenza Epstein–Barr virus commonly could cause olfactory dysfunction (OD) by leading an inflammation in the olfactory mucosa resulting in rhinorrhea. Instead, COVID-19 seems to cause an atypical OD as it develops without rhinorrhea or nasal congestion (36).

In 2007, Suzuki et al. identified that coronavirus could be associated with anosmia, and he already speculated that nasal inflammation and related obstruction were not the only etiological factors underlying the OD in viral infection (37). As well-reported in the literature, HCoV could infect peripheral nerve terminals, using the trans-synaptic transfer to access the CNS (36, 38, 39)

In our preliminary observation, the administration of Dox 200 mg once daily seems to improve respiratory symptoms and anosmia under Dox treatment in six patients completely recover after only 2 days of treatment. From our experience, it seems reasonable to continue the treatment at least 8 days. The mean patients' age was 35.8 ± 6.8 years, and 4 (66.7%) were females. One patient reported anosmia as the only COVID-19 manifestation; instead of the other five patients who complained about the loss of smell, in which it appeared 5–7 days after mild fever, dry cough, and malaise. The average time of the recovery COVID-19-linked anosmia after the administration of Dox in these patients was 2.5 ± 0.5 days. We noticed a sudden improvement in all symptoms after the administration of Dox, but our most exciting insight is about the rapid recovery of the smell.

Unlike olfactory sensory neurons (OSNs), nasal epithelium, which includes the respiratory and olfactory epithelium (OE) expresses high levels of ACE2 (40). SARS-CoV-2 seems to target non-neural cell types in the peripheral olfactory system rather than directly enter OSNs, and it seems to be enough to generate cascading damage that could lead to the impairment of OSNs function altering the odor transduction which takes place on their cilia (40). The short-term COVID-19-linked anosmia reported in our experience supports the hypothesis that SARS-CoV2 affects the OE, which can quickly renew and recover following viral clearance (41). The average time to restore the sense of smell, most commonly reported in the literature, lasts from 1–8 days (36), if SARS-COV-2 could directly damage OSNs, recovery should take longer (42). Besides ACE2, Brann et al. also revealed that a cell-surface receptor, CD147, could play a role mediating SARS-CoV-2 cell entry (40). The expression of CD147 is detected in ciliated and goblet cells in the human nasal mucosa (43). Previous reports have shown that Dox has a significant inhibitory effect on CD147 expression (44, 45). Further studies are needed at present to define better if Dox has the ability to inhibiting viral entry by reduced CD147 expression levels. Moreover, thanks to its immunomodulatory and anti-inflammatory properties, Dox could limit the pro-inflammatory state induced by the glial cells activated by the neurotropic virus, ensuring proper epithelial reconstitution in the OE (46, 47). Given the possibility that COVID-19 occurs with the loss of smell and the evidence that corticosteroid may worsen the infection (48), Prof. Claire Hopkins, the British Rhinological Society president, recently suggested avoiding the use of these drugs in the therapeutic approach to the new-onset anosmia during the COVID-19 pandemic, especially if unrelated to previous head trauma or nasal pathology (48).

We are perfectly aware that there is a need for stronger evidence, but our article would intend to underline the importance of considering smell loss as a common symptom of COVID-19, supporting the rationale to treat such patients with Dox based on its interesting antiviral properties.

## Author Contributions

CB and DB: contributed equally to this manuscript, wrote the article, and reviewed the final version. AL and EB: review and editing of the final manuscript. All authors reviewed the manuscript and agreed with its content.

## Conflict of Interest

The authors declare that the research was conducted in the absence of any commercial or financial relationships that could be construed as a potential conflict of interest.
